# Intracellular Analysis of the Interaction between the Human Papillomavirus Type 16 E6 Oncoprotein and Inhibitory Peptides

**DOI:** 10.1371/journal.pone.0132339

**Published:** 2015-07-07

**Authors:** Christina Stutz, Eileen Reinz, Anja Honegger, Julia Bulkescher, Johannes Schweizer, Katia Zanier, Gilles Travé, Claudia Lohrey, Karin Hoppe-Seyler, Felix Hoppe-Seyler

**Affiliations:** 1 Molecular Therapy of Virus-Associated Cancers (F065), Program Infection and Cancer, German Cancer Research Center (DKFZ), 69120 Heidelberg, Germany; 2 Arbor Vita Corporation, Fremont, CA 94085, USA; 3 Institut de Recherche de l’École de Biotechnologie de Strasbourg (IREBS), 67412 Illkirch, France; Albert Einstein College of Medicine, UNITED STATES

## Abstract

Oncogenic types of human papillomaviruses (HPVs) cause cervical cancer and other malignancies in humans. The HPV E6 oncoprotein is considered to be an attractive therapeutic target since its inhibition can lead to the apoptotic cell death of HPV-positive cancer cells. The HPV type 16 (HPV16) E6-binding peptide pep11, and variants thereof, induce cell death specifically in HPV16-positive cancer cells. Although they do not encompass the LxxLL binding motif found in cellular HPV16 E6 interaction partners, such as E6AP, the pep11 variants strongly bind to HPV16 E6 by contacting the recently identified E6AP binding pocket. Thus, these peptides can serve as prototype E6-inhibitory molecules which target the E6AP pocket. We here analyzed their intracellular interaction with HPV16 E6. By comprehensive intracellular binding studies and GST pull-down assays, we show that E6-binding competent pep11 variants induce the formation of a trimeric complex, consisting of pep11, HPV16 E6 and p53. These findings indicate that peptides, which do not contain the LxxLL motif, can reshape E6 to enable its interaction with p53. The formation of the trimeric HPV16 E6 / peptide / p53 complex was associated with an increase of endogenous HPV16 E6 protein amounts. Yet, total cellular p53 amounts were also increased, indicating that the E6 / E6AP-mediated degradation of p53 is blocked. These findings suggest that inhibition of oncogenic activities by targeting the E6AP pocket on HPV16 E6 could be a strategy for therapeutic intervention.

## Introduction

Cervical cancer is a major malignancy in women worldwide [1]. Almost all cases (>99%) are associated with high-risk human papillomaviruses (HPVs), most prominently HPV type 16 (HPV16), which alone accounts for approximately 50% of all cervical cancer cases [2]. The cooperative activities of the viral E6 and E7 oncoproteins are essential for the initiation and maintenance of the malignant phenotype of HPV-positive tumor cells. In this scenario, the E7 protein stimulates cell proliferation and the E6 protein has a major role in counteracting the reactive induction of apoptosis towards this abnormal growth stimulus [3,4].

At the biochemical level, the E6 protein interacts with the cellular E3 ubiquitin ligase E6AP [5]. This alters the substrate specificity of E6AP and mediates the binding of E6 / E6AP to p53, resulting in a trimeric complex. E6AP can subsequently ubiquitinate p53, which in turn is degraded by the 26S proteasome [6,7]. In addition to this well characterized trimeric E6 / E6AP / p53 complex formation, studies reported E6AP-independent binding of high-risk HPV E6 proteins to p53 [8], E6AP-independent p53 degradation induced by high-risk E6 *in vitro* [9,10], and E6AP-independent inactivation of p53 in transgenic mice [11]. These findings raise the possibility that the E6 oncoprotein might also directly or indirectly interact with p53 in the absence of E6AP.

By various experimental approaches, it has been shown that blocking E6 can lead to the induction of apoptosis in HPV-positive cancer cells [12–16]. This suggests that the targeted inhibition of E6 represents a promising approach to develop specific therapeutic strategies to combat HPV-positive cancers, and possibly HPV-positive preneoplasias [4,17,18]. Thus, it is important to explore the interaction of inhibitory molecules with E6, and the resulting biological consequences.

We here study the interaction between HPV16 E6 and its inhibitory 15-mer peptide “pep11”, that was identified by screening a randomized peptide expression library for E6-binding molecules [16]. Pep11, as well as its solubility-optimized variants pep11* and pep11**, contain a novel E6-binding motif which is different from the known LxxLL motif found in natural interaction partners of HPV16 E6, such as in E6AP [16,19]. In contrast to a peptide corresponding to the E6-binding domain of E6AP [13] (here termed “E6APpep”), pep11 and its variants not only bind to HPV16 E6, but also efficiently induce apoptosis, specifically in HPV16-positive cells [16,19]. We found that pep11** binds with high affinity to the E6AP-binding pocket [19], a structure which has been recently elucidated by X-ray analysis, with E6APpep bound in the pocket [20]. The binding of pep11** or E6APpep to the E6AP binding pocket involves many identical amino acid residues of HPV16 E6, but also shows distinct differences, with few amino acids differentially contributing to the two interactions [19].

Thus, pep11** represents a prototype E6-inhibitory molecule acting via the E6AP binding pocket of HPV16 E6. In this work, we show that pep11** co-localizes with HPV16 E6 and that its expression leads to increased levels of HPV16 E6. Moreover, binding to pep11 variants enables HPV16 E6 to form trimeric E6 / pep11 / p53 complexes. These results provide the first experimental evidence that E6 can be stabilized and reshaped for complex formation with p53 by peptides or proteins which do not contain the LxxLL sequence. Moreover, despite the increase of E6 amounts, overall p53 concentrations are also increased. This indicates that E6-binding pep11 variants block E6-mediated p53 degradation by capturing E6 in trimeric E6 / pep11 / p53 complexes. Taken together, these findings suggest that targeting the E6AP binding pocket by inhibitory molecules could represent a therapeutic strategy, albeit these molecules can bring E6 and p53 in close vicinity.

## Materials and Methods

### Cell culture, plasmids and transfections

HeLa (HPV18-positive), SiHa and MRI-H186 (both HPV16-positive) cervical carcinoma cells were obtained from the tumor bank of the German Cancer Research Center, Heidelberg (HeLa, MRI-H186) or from the American Tissue Culture Collection (SiHa). Cells were cultivated in DMEM (HeLa, SiHa) or RPMI (MRI-H186), supplemented with 10% fetal bovine serum (Gibco Life Technologies, Carlsbad, CA, USA), 2 mM L-glutamine, 100 U/ml penicillin and 100 μg/ml streptomycin (Sigma-Aldrich, St. Louis, MO, USA). P53-null H1299/K3 cells (kindly provided by Prof. Martin Scheffner, University of Konstanz, Germany) are H1299 cells in which endogenous E6AP expression is reduced by RNA interference [21]. H1299/K3 cells were cultivated in DMEM, supplemented with 4 μg/ml puromycin, 10% fetal bovine serum (Gibco Life Technologies), 2 mM L-glutamine, 100 U/ml penicillin and 100 μg/ml streptomycin (Sigma-Aldrich). Peptides pep11*, pep11**, pep11’, E6APpep, pep11*m, pep11**m and pep11’m were either expressed in fusion to the GAL4 DNA binding domain (GAL4-BD) from vector pBIND (Promega, Madison, WI, USA) or in fusion to human recombinant green fluorescent protein (hrGFP; Stratagene, Heidelberg, Germany) from pCEP4 (Invitrogen), as previously described [16]. Wildtype or mutant flag-tagged E6 proteins and p53 were expressed in fusion to the VP16 activation domain (VP16-AD) from vector pACT (Promega) [19]. HPV16 E6 was expressed in fusion to GAL4-BD or mCherry from vector pBIND or pcDNA3, respectively. Plasmids pNCMV16E6, pNCMV11E6 (kind gifts of Prof. Karl Münger, Tufts University, Boston, USA) and p53wt have been described before [16,22]. Plasmids were transfected by calcium phosphate co-precipitation as described [16] or by Fugene HD (Roche Diagnostics, Penzberg, Germany), following the protocol provided by the supplier. To each transfection, equal amounts of β-Galactosidase expressing plasmid pCMV-Gal [23] were added to allow comparison of transfection efficiencies.

### Mammalian two-hybrid assays

The CheckMate system (Promega, Madison, WI, USA) was used as a basis for intracellular interaction studies, with modifications as described in the text. For all experiments, both pBIND and pACT fusion constructs were transfected into HeLa, along with the GAL4-responsive luciferase reporter construct pG5luc and internal standard pCMV-Gal. Two days after transfection, cells were harvested. Luciferase activities were determined as duplicates in three independent experiments using a Mithras LB943 Monochromator Multimode Reader (Berthold, Bad Wildbad, Germany) and normalized for β-Galactosidase activities.

### Confocal microscopy

HeLa cells grown on coverslips were fixed two days post transfection with paraformaldehyde (4% in PBS) and permeabilized with PBS/0.2% Triton X-100. Immunostaining with primary antibodies anti-hrGFP 240241 (Agilent Technologies, Santa Clara, CA, USA) or anti-vimentin clone V9 (Sigma-Aldrich) in PBS / 0.3% BSA was done overnight at 4°C, followed by incubation with anti-mouse Cy5 A10524 (Invitrogen, Karlsruhe, Germany) together with 4’,6-Diamidino-2-phenylindole (100 ng/ml) (Roche Diagnostics) for 1 hour. Three-dimensional images were obtained by confocal microscopy with a Zeiss LSM 710 ConfoCor 3 microscope using a 63 oil immersion objective. Where indicated, cells were treated with Nocodazole (10 μg/ml) (Calbiochem, San Diego, CA, USA) for 24 hours before fixation.

### Peptide synthesis

Peptides pep11** (sequence: KEKEEYNSNCSCIACIGLI) and pep11**m (KEKEEYNSNSSSIASIGLI) [16] were chemically synthesized by the Peptide Specialty Laboratory (Heidelberg, Germany). Crude peptides were purified by HPLC and checked by mass spectrometry. Peptides were dissolved in DMSO to obtain 10 mM solutions and stored at -20°C.

### Immunoblot analyses

Cellular protein was extracted in RIPA buffer (10 mM Tris–HCl pH 7.5, 150 mM NaCl, 1 mM EDTA, 1% NP40, 0.5% sodium deoxycholate, 0.1% SDS), two days after transfection. Detergent-insoluble protein fractions were prepared as described previously [24]. Approximately 15 μg of protein were separated by a NuPage 4–12% Bis-Tris protein gel (Thermo Fisher Scientific, Waltham, MA, USA) and subsequently electrotransferred to an Immobilon-P membrane (Millipore, Bedford, MA, USA) using the Trans-Blot Semi-Dry Transfer Cell (Bio-Rad, München, Germany). Membranes were blocked with 5% skim milk powder (Saliter, Obergrünzburg, Germany) and 1% bovine serum albumin (BSA, Sigma-Aldrich) in PBS-T (PBS, 0.2% Tween-20) for 1 h at room temperature. Membranes were incubated with primary antibodies overnight at 4°C in PBS-T/5% skim milk powder/1% BSA, followed by incubation with the corresponding HRP-conjugated secondary antibody for 1 hour at room temperature. Proteins were visualized using ECL Prime Western Blotting Detection Reagent (GE Healthcare, Buckinghamshire, UK). Images were monitored using the Fusion SL Gel Detection System (Vilber Lourmat, Marne-la-Vallée, France). The following primary antibodies were used: anti-alpha-tubulin #CP06 (Calbiochem), anti-E6AP E8655, anti-flag F7425 (Sigma-Aldrich), anti-GAL4DBD sc-577 (Santa Cruz Biotechnology, Dallas, TX, USA), anti-GST 06–332 (Upstate, Merck KGaA, Darmstadt, Germany), anti-HPV16E6 clone 849 (Arbor Vita Corporation, Fremont, CA, USA), anti-hr-GFP [16], anti-p21 CatP06 (Calbiochem) and anti-p53 DO-1 (Santa Cruz Biotechnology).

### Colony formation assays

Cell lines transfected with hrGFP-peptide expression vectors were selected for the presence of pCEP4 by hygromycin B treatment. Colonies were fixed and stained with formaldehyde-crystal violet after 7 to 14 days of selection.

### GST Pull-down assays

H1299/K3 cells transfected with p53wt were harvested 24 hours post transfection with sterile-filtrated E1A lysis buffer (250 mM NaCl, 0.1% Triton X-100, 50 mM Hepes, pH7.4, 10 mM MgCl_2_, protease inhibitor cocktail (Complete Mini EDTA-free; Roche Diagnostics)). Lysates were chilled on ice for 30 minutes and then centrifuged at 10.000 *g* and 4°C for 15 minutes. A 30 μl-sample of 50% slurry of glutathione Sepharose 4B beads (GE Healthcare) was equilibrated in E1A lysis buffer by washing the beads 5 times with buffer. Equimolar amounts of GST-HIS (6 μg) or GST-HPV16 E6 (10.5 μg) were added to the beads and filled up with E1A buffer to 500 μl. GST-HIS or GST-HPV16 E6 were immobilized on the beads by rotation for 2–4 hours at 4°C. After 1 hour, 1 μl of a 10 mM solution of the synthetic peptides or DMSO was added to the beads. Pre-cleared lysate was generated as follows: lysate was incubated for 2 hours with GST-HPV16 E6 protein-bound beads, followed by centrifugation at 4°C and 500 *g* for 5 minutes. H1299/K3 cell-lysates (0.15 mg), either pre-cleared or uncleared, were added to the GST-HIS or GST-HPV16 E6 protein-bound beads and filled up with E1A buffer to 1 ml end volume, followed by incubation at 4°C overnight. Beads were washed five times with 800 μl E1A buffer. Subsequently, the beads were resuspended in an appropriate volume of E1A buffer and 4X protein sample buffer (0.47 M Tris pH 6.7, 2% (m/v) SDS, 16% (v/v) glycerol, 6% (v/v) 2-mercaptoethanole, 0.4% (m/v) bromphenolblue), and subjected to immunoblot analysis.

### Statistical analyses

Statistical significance of differences in measured RLAs between the samples, as detailed in the text, was evaluated by a two-sided paired t-test using the Sigma Plot software (Systat Software Inc., San Jose, CA, USA).

## Results

### Intracellular co-localization of HPV16 E6 and pep11**

Endogenous HPV16 E6 protein levels are believed to be relatively low in HPV16-positive cancer cells and, in our hands, are not amenable to immunofluorescence-based detection with available antibodies. In order to visualize the intracellular interaction between pep11** and HPV16 E6, we therefore co-expressed mCherry-HPV16 E6 together with either hrGFPpep11** or -pep11**m in HeLa cells.

**Fig 1:**
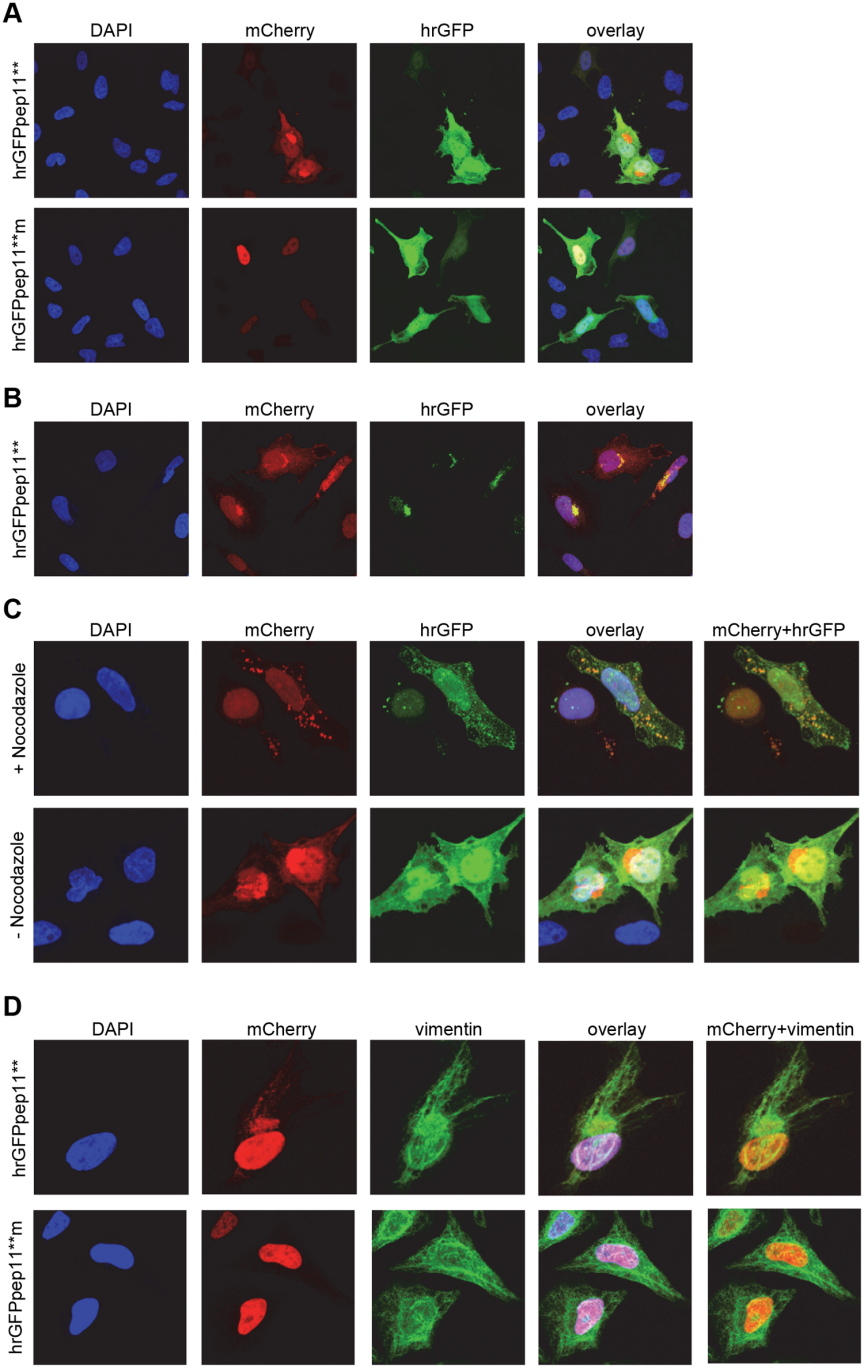
Intracellular co-localization of pep11** and HPV16 E6. (A) mCherry-HPV16 E6 was co-expressed in HeLa cells with either hrGFPpep11** (upper panel) or hrGFP-pep11**m (lower panel). (B) Immunostaining for hrGFP upon co-expression of mCherry-HPV16 E6 with hrGFPpep11** in HeLa cells. (C) Upper panel: Nocodazole treatment of HeLa cells upon co-expression of mCherry-HPV16 E6 with hrGFPpep11**. Lower panel: Untreated control cells. (D) Immunostaining for vimentin in HeLa cells co-expressing mCherry-HPV16 E6 with either hrGFPpep11** (upper panel) or hrGFP-pep11**m (lower panel).

Previous studies have indicated that ectopically expressed HPV16 E6 locates to the nucleus [25,26]. Consistently, upon co-expression of HPV16 E6 with the E6-binding defective mutant peptide pep11**m, HPV16 specific E6 signals were primarily nuclear whereas pep11**m was distributed throughout the cell (Fig 1A, lower panel). Interestingly, however, upon co-expression of HPV16 E6 with the E6-binding competent pep11** peptide, a substantial proportion of E6 was shifted to a perinuclear compartment (Fig 1A, upper panel). Surprisingly, we did not observe a signal at this site for pep11**-hrGFP in the hrGFP-channel (Fig 1A, upper panel). Since the fluorescence of GFP can depend on its solubility [27], we alternatively applied an anti-hrGFP antibody for pep11**-hrGFP detection, and this approach revealed both pep11** and HPV16 E6 to co-localize in perinuclear structures (Fig 1B).

These findings raised the question whether HPV16 E6 and hrGFPpep11** may co-localize in aggresomes which are perinuclear compartments that harbor protein aggregates [28,29]. To test this hypothesis, we treated the cells with nocodazole, a microtubule depolymerizing agent which prevents aggresome formation [28,29]. In nocodazole-treated cells, hrGFPpep11** and HPV16 E6 co-localized in distinct foci within the cytosol, but no longer in the perinuclear space (Fig 1C). Moreover, the perinuclear pep11** / HPV16 E6 accumulations were surrounded by a vimentin cage (Fig 1D), as typical for aggresomes [28,29]. In conclusion, these data indicate that pep11** and HPV16 E6 bind intracellularly upon ectopic expression and are translocated into aggresomes.

### Ectopic co-expression of HPV16 E6 with HPV 16 E6-binding pep11 variants leads to increased E6 protein levels

Next, we performed immunoblot analyses after co-expression of HPV16 E6 together with E6-binding competent peptides. In addition to pep11**, we also applied peptide pep11’, a more soluble variant of pep11**, that shares the same biological features (specific binding to HPV16 E6 and to certain mutants of HPV18 and HPV31 E6, inhibition of HPV16-positive cells in colony formation assays, S1 Fig). By co-expressing HPV16 E6 together with either hrGFP-pep11**, -pep11**m, -pep11’, -pep11’m or -E6APpep, we addressed two questions: (i) does expression of E6-binding pep11 variants result in a shift of HPV16 E6 from the detergent-soluble to the detergent-insoluble protein fraction, as would be expected if pep11 / HPV16 E6 complexes are translocated into aggresomes [28,29]? (ii) Do HPV16 E6 protein levels change upon co-expression of E6-binding pep11 variants? Increases in E6 levels have been described upon expression of E6AP [30] or peptides carrying the E6APpep motif [31].

**Fig 2:**
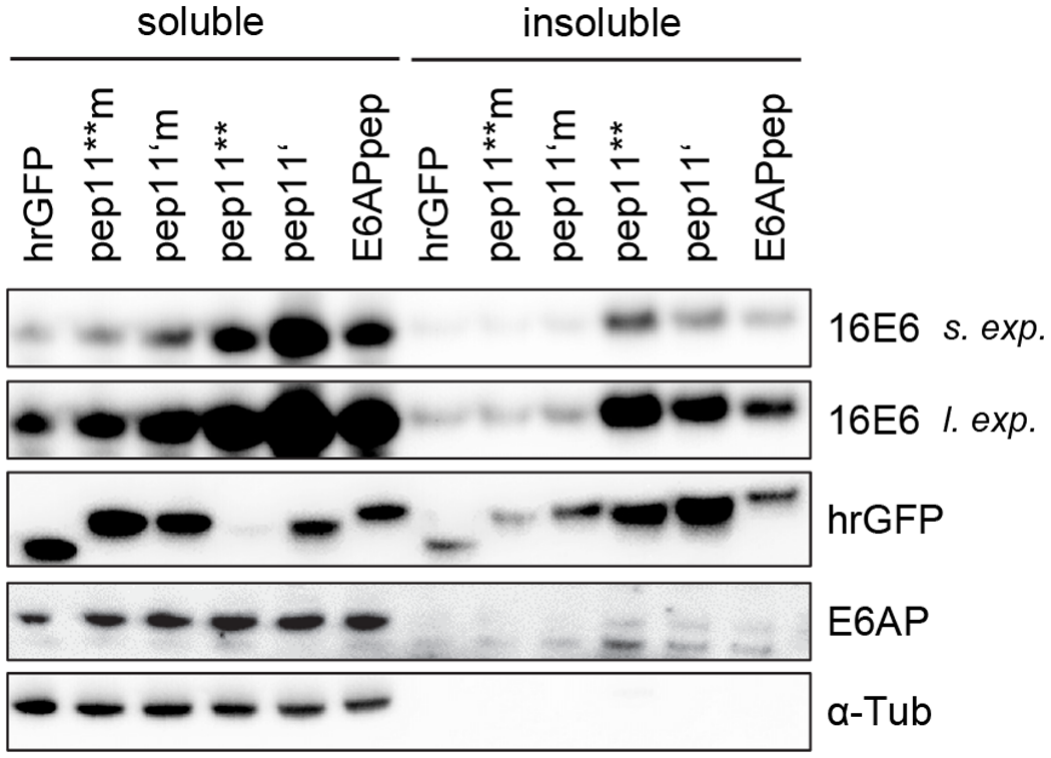
Immunoblot analyses of HPV16 E6, upon ectopic co-expression with E6-targeting peptides. Expression of HPV16 E6 in HeLa cells together with either hrGFP-linked control peptides pep11**m or pep11’m (both E6-binding defective), or pep11**, pep11’ or E6APpep (all E6-binding competent). Soluble and insoluble protein fractions are indicated. Loading of protein extracts was normalized for equal transfection efficiencies, as determined by activities of a co-transfected β-galactosidase expression vector. Expression levels of endogenous E6AP and of individual peptide-hrGFP fusion proteins are indicated. α-Tub, α-tubulin; s. exp., short exposure; l. exp., long exposure.

Consistent with the immunofluorescence data, the HPV16 E6-binding competent pep11** and pep11’ peptides induced a readily detectable shift of HPV16 E6 protein from the soluble into the insoluble protein fraction (Fig 2) when compared with the corresponding E6-binding defective pep11**m and pep11’m controls. This was also observed for the ectopic co-expression of HPV16 E6 with E6APpep. Notably, and alike E6APpep, both pep11** and pep11’ induced a clear increase in HPV16 E6 protein levels in both fractions when compared to the respective E6-binding defective control peptides (Fig 2). By contrast, the amount of endogenous E6AP protein was not affected.

### Intracellular expression of HPV16 E6-binding pep11 variants leads to increased endogenous E6 protein levels

**Fig 3:**
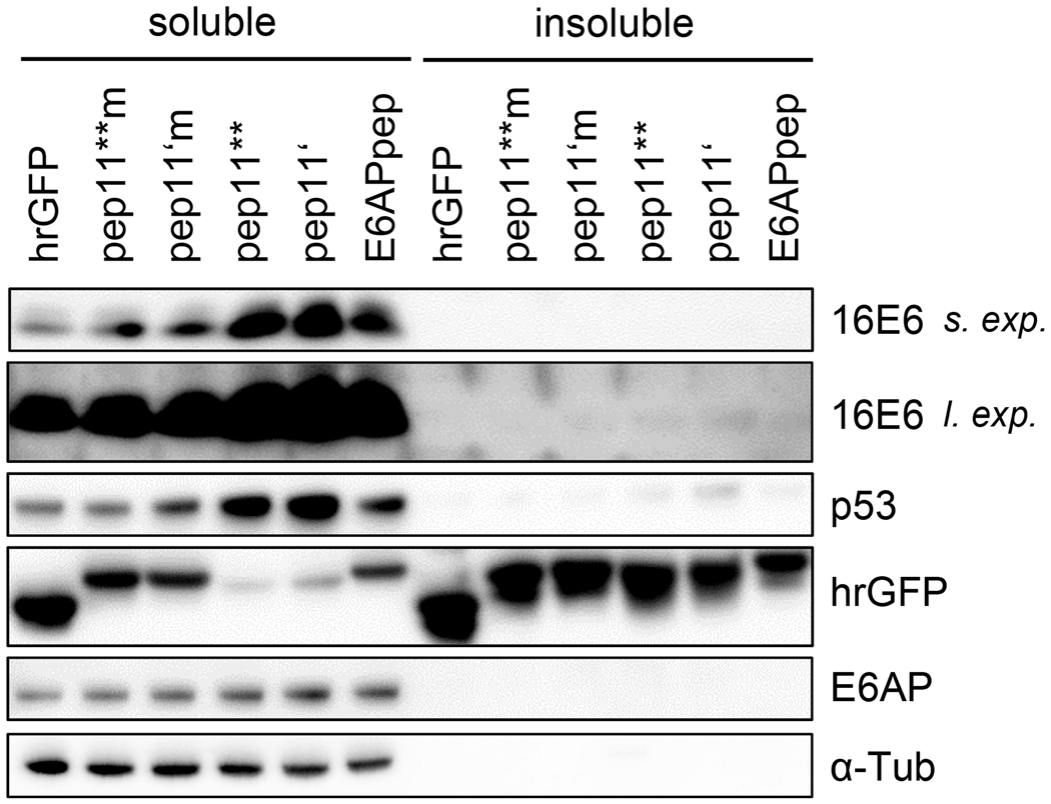
Immunoblot analyses of endogenous HPV16 E6, upon intracellular expression of E6-targeting peptides. Expression of either hrGFP-linked peptides pep11**m or pep11’m (both E6-binding defective), or pep11**, pep11’ or E6APpep (all E6-binding competent) in HPV-16 positive MRI-H186 cells. Soluble and insoluble protein fractions are indicated. Loading of protein extracts was normalized for equal transfection efficiencies, as determined by the activities of a co-transfected β-galactosidase expression vector. Expression levels of endogenous E6AP, of p53, and of individual peptide-hrGFP fusion proteins are indicated. α-Tub, α-tubulin; s. exp., short exposure; l. exp., long exposure.

Next, we extended these investigations and analyzed endogenous E6 protein levels in HPV16-positive cancer cells. For this, hrGFP-linked E6APpep, pep11** or pep11’, and the E6-binding defective mutants pep11**m or pep11’m, were expressed in HPV16-positive MRI-H186 cells. Under these experimental conditions, only little E6 was detectable in the insoluble protein fraction, even after long exposure times of the immunoblots. Importantly, and as seen in the ectopic co-expression experiments (Fig 2), endogenous HPV16 E6 levels increased upon intracellular pep11** or pep11’ expression, as also observed upon expression of E6APpep (Fig 3). This increase of endogenous E6 amounts upon expression of E6-binding competent pep11 variants is also detectable in other HPV16-positive cervical cancer cells, as shown for SiHa cells (S2 Fig). Taken together, these findings indicate that, alike E6APpep-containing peptides [31], binding of pep11 variants to HPV16 E6 may stabilize the E6 protein. These results furthermore raise the question whether the E6-binding competent pep11 variants may induce a conformational change of HPV16 E6 that enables complex formation with p53, even though they show distinct binding differences compared to the HPV16 E6 / E6APpep interaction and do not contain the LxxLL motif [16,19].

### Intracellular bridging of HPV16 E6-binding pep11 variants and p53 by HPV16 E6

**Fig 4:**
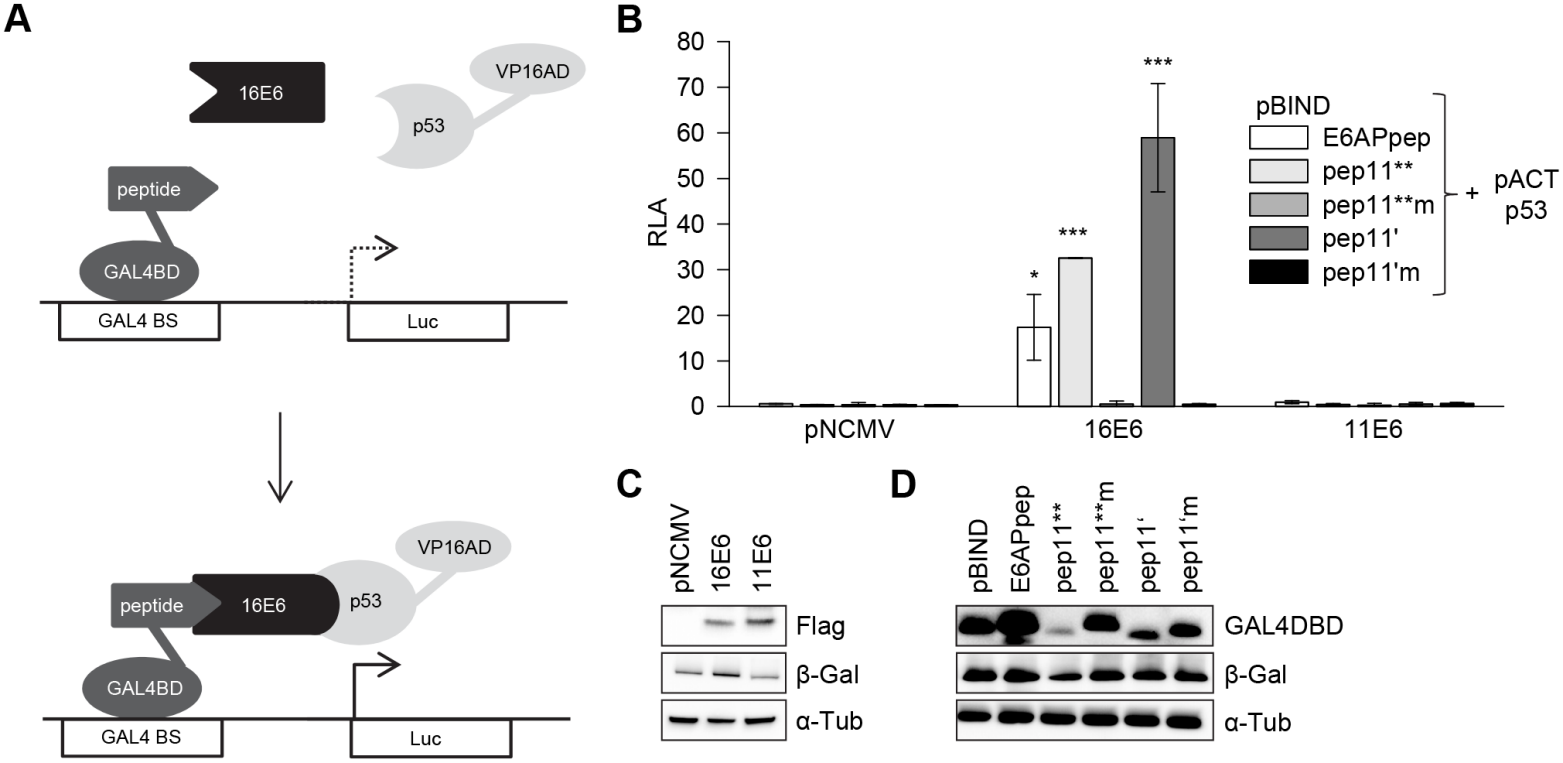
HPV16 E6 mediating intracellular formation of a trimeric complex with E6-binding peptides and p53. (A) Schematic illustration of the employed modified mammalian two hybrid assay. Upper panel: Individual peptides are linked to GAL4-BD, p53 is linked to VP16-AD, E6 proteins are expressed from a co-transfected expression vector. Lower panel: Trimeric complex formation is detected by activation of the luciferase reporter. GAL4 BS, GAL4 binding sites. (B) Co-expression of individual peptides linked to the GAL4-BD, as indicated, together with p53-VP16-AD and HPV16 E6 or HPV 11 E6, respectively. Empty expression vector pNCMV served as negative control. Indicated are relative luciferase activities (RLA) above those of control-transfected cells, expressing the corresponding peptide-GAL4-BD fusions together with the co-transfected empty vectors pACT and pNCMV; values are arbitrarily set at 1.0. Results were obtained from three individual experiments, each performed in duplicates. Standard deviations are indicated. Asterisks above the columns indicate significant differences above those of cells expressing the corresponding peptides linked to GAL4-BD together with p53-VP16-AD, in the absence of E6 (co-transfected empty vector pNCMV), with p-values of ≤0.001 (***) and ≤0.05 (*). (C) Immunoblot analyses of expression levels of Flag-tagged HPV16 E6 and HPV11 E6 and of (D) individual peptides linked to GAL4-DB. Loading of protein extracts was normalized for equal transfection efficiencies, as determined by a co-transfected β-galactosidase expression vector. β-Gal, β-galactosidase; α-Tub, α-tubulin.

Therefore, we tested the ability of E6 to undergo trimeric complex formation with p53, in the presence of E6-binding competent and E6-binding defective pep11 variants. To study this under intracellular conditions, we performed modified mammalian two-hybrid analyses in HeLa cells, by co-expressing: (i) individual peptides (E6APpep, pep11**, pep11**m, pep11’, pep11’m) linked to the GAL4 DNA binding domain (GAL4BD), (ii) p53 fused to the VP16 transactivation domain (VP16AD), and (iii) flag-tagged HPV16 or HPV11 E6 protein. If a trimeric complex is formed by these components, E6 could bridge the peptides linked to the GAL4BD with p53 fused to VP16AD, resulting in the activation of a co-transfected luciferase reporter plasmid under transcriptional control of GAL4 binding sites (Fig 4A).

In this setting, none of the tested peptides interacted with p53 in the absence of E6 (Fig 4B). In contrast, co-expression of HPV16 E6, together with E6APpep and p53, resulted in the activation of the reporter plasmid (Fig 4B). These properties are consistent with the ability of E6APpep to reshape HPV16 E6 and enable its complex formation with p53 [31].

Importantly, however, we also observed that expression of HPV16 E6 together with pep11**, or pep11’, and p53 activated the luciferase reporter (Fig 4B). No stimulation of the reporter plasmid was observed when exchanging pep11** and pep11’ with the respective E6-binding defective pep11**m and pep11’m controls. In addition, no stimulation of the reporter plasmid was observed for the co-expression of pep11** or pep11’ with p53 and comparable amounts of low-risk HPV11 E6, instead of HPV16 E6 (Fig 4B and 4C). This is consistent with the observation that pep11 does not bind to HPV11 E6 [16]. Taken together, these results provide evidence at the intracellular level that, alike E6APpep, binding of pep11** or pep11’ restructures HPV16 E6 in a way that it allows complex formation with p53. Notably, stimulation of the reporter plasmid by the pep11** / HPV16 E6 / p53 (33-fold) and pep11’ / HPV16 E6 / p53 (59-fold) complexes was stronger than that observed for the E6APpep / HPV16 E6 / p53 complex (17-fold), although E6APpep was expressed at higher quantities than the E6-binding competent pep11 variants (Fig 4D).

### Intracellular bridging of HPV16 E6 and p53 by E6-binding pep11 variants

**Fig 5:**
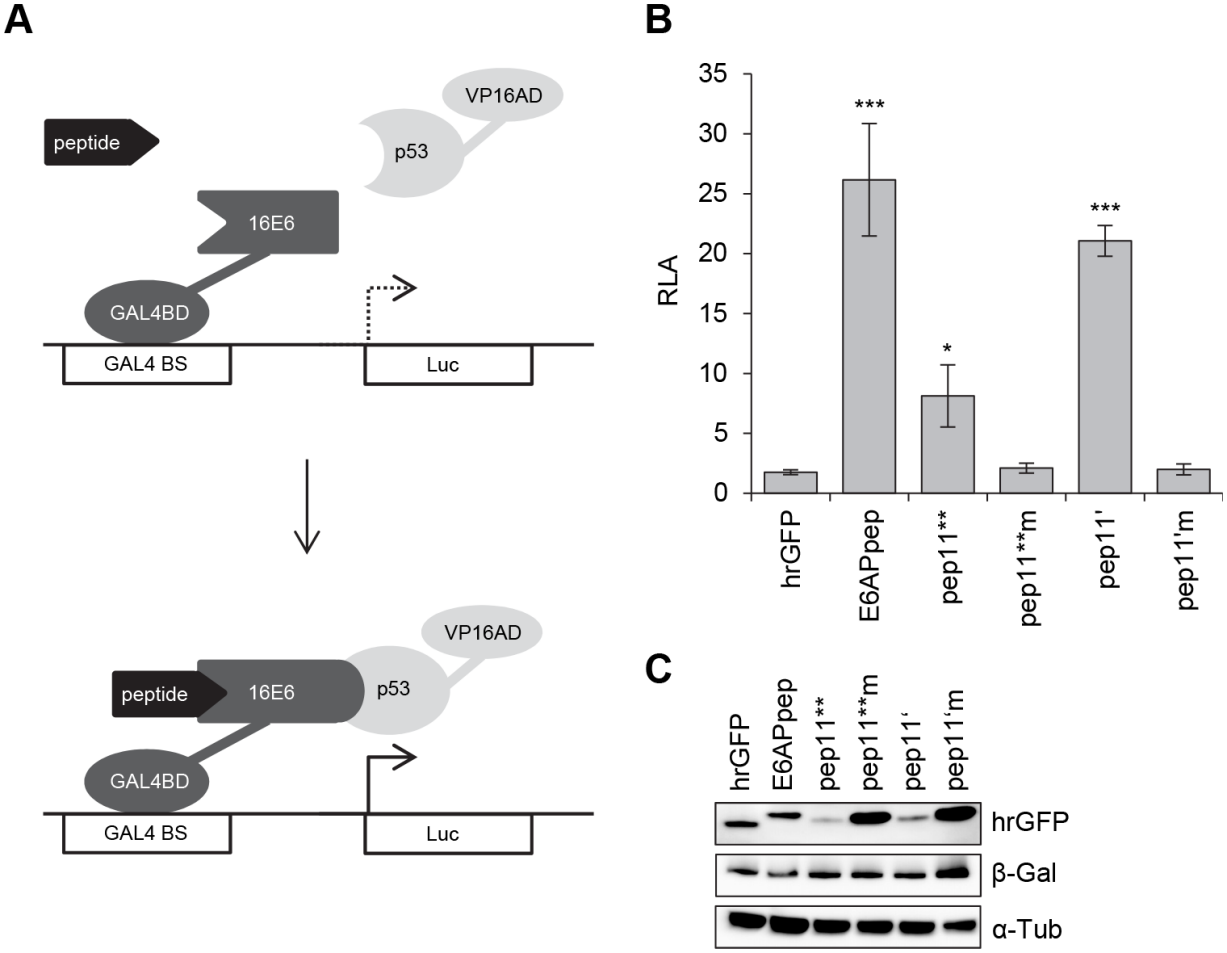
E6-binding peptides mediating intracellular formation of a trimeric complex with HPV16 E6 and p53. (A) Schematic illustration of the employed modified mammalian two hybrid assay. Upper panel: HPV16 E6 is linked to GAL4-BD, p53 is linked to VP16-AD, individual peptides are expressed from a co-transfected expression vector, in fusion with hrGFP. Lower panel: Trimeric complex formation is detected by activation of the luciferase reporter. GAL4 BS, GAL4 binding sites. (B) Expression of HPV16 E6-GAL4-BD with p53-VP16-AD, together with hrGFP-linked peptides, as indicated, or, as negative control, hrGFP alone. Shown are relative luciferase activities (RLA) above those of control-transfected cells, expressing HPV16 E6-GAL4-BD fusions together with the co-transfected basic vectors pACT and pCEP4hrGFP; values are arbitrarily set at 1.0. Results were obtained from three individual experiments, each performed in duplicates. Standard deviations are indicated. Asterisks above the columns indicate significant differences above those of cells expressing HPV16 E6-GAL4-BD together with p53-VP16-AD, in the absence of the peptides (co-transfected basic vector pCEP4hrGFP), with p-values of ≤0.001 (***) and ≤0.05 (*). (C) Immunoblot analyses of expression levels of individual peptides linked to hrGFP. Loading of protein extracts was normalized for equal transfection efficiencies, as determined by a co-transfected β-galactosidase expression vector. β-Gal, β-galactosidase; α-Tub, α-tubulin.

The findings using the modified mammalian two-hybrid system described above do not rule out that HPV16 E6 alone may interact with p53, in the absence of co-expressed E6-binding peptides. Thus, in the next set of experiments, we co-expressed HPV16 E6 fused to GAL4BD and p53 linked to VP16AD, in the presence of E6-binding competent and E6-binding defective peptides (Fig 5A). When HPV16 E6 and p53 were co-expressed in this setting in the absence of the peptides, we observed only a marginal activation (1.7-fold) of the luciferase reporter above background levels, indicating that HPV16 E6 and p53 alone only very weakly interact intracellularly, if at all (Fig 5B). In contrast, the additional co-expression of E6APpep, pep11** and pep11’ led to a strong activation of the luciferase reporter (Fig 5B). This was not observed for the E6-binding defective control peptides pep11**m or pep11’m, although they were expressed at higher levels than their corresponding E6-binding competent pep11 variants (Fig 5C). These findings corroborate that, alike E6APpep, E6-binding pep11 variants can mediate HPV16 E6 complex formation with p53. Neither of the peptides (Fig 4) nor HPV16 E6 alone (Fig 5) efficiently interacted with p53, indicating that a peptide-induced conformational change of HPV16 E6 is required for the intracellular formation of this trimeric complex.

### GST-Pulldown analyses of pep11**-induced binding of HPV16 E6 to p53

**Fig 6:**
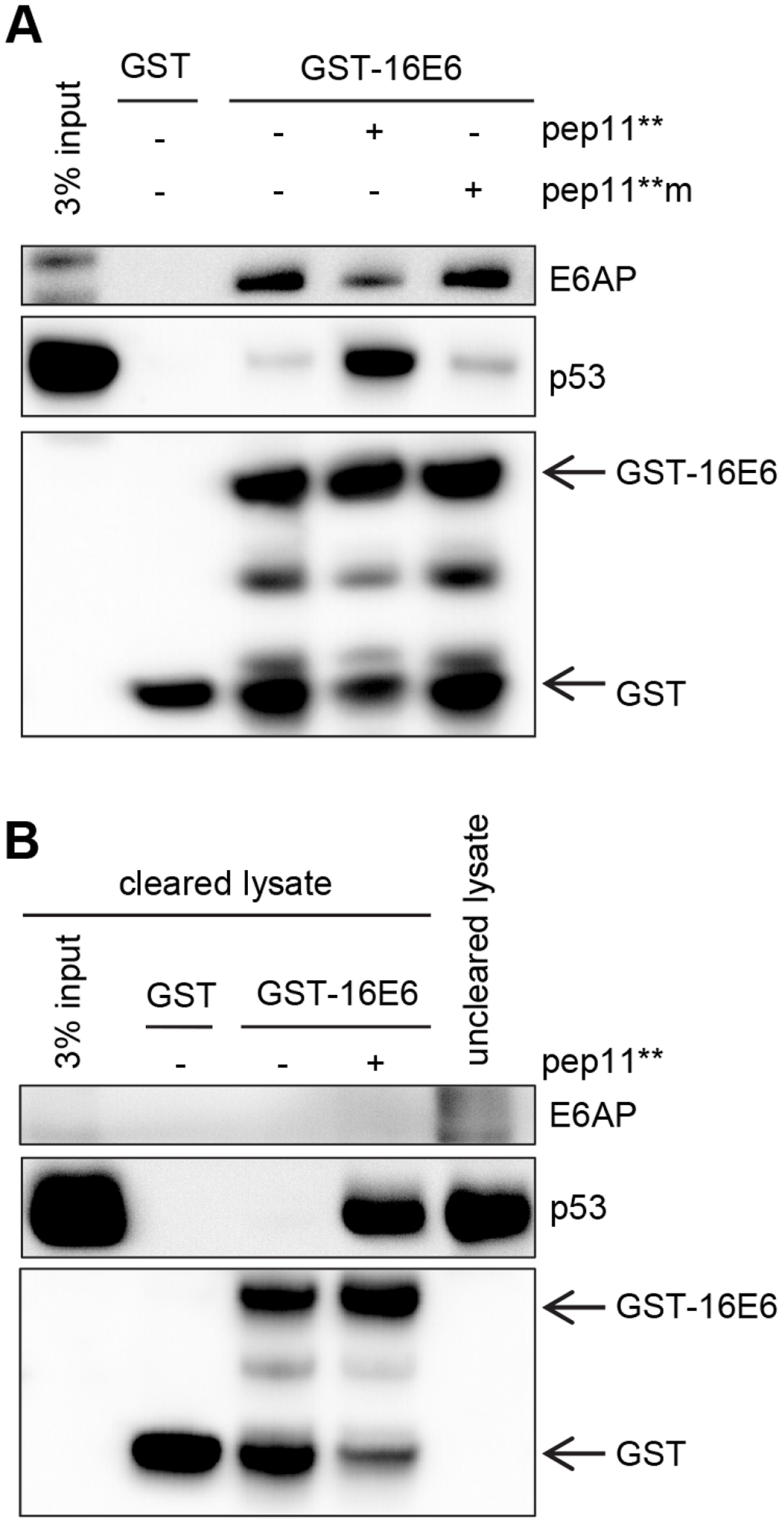
Trimeric complex formation between HPV16 E6, p53 and pep11** or E6AP, in GST pull-down analyses. (A) p53 was expressed in H1299/K3 cells and pull-down assays were performed with GST-tagged HPV16 E6 (GST-16E6) in the presence of no peptide, synthetic pep11** or synthetic pep11**m. E6AP and p53 bound to GST-16 E6 are indicated. (B) The H1299/K3 lysate was pre-cleared from endogenous E6AP protein by pre-incubation with GST-16 E6. E6AP and p53 bound to GST-16 E6 are indicated.

To further confirm the complex formation between HPV16 E6, pep11** and p53 by an independent experimental approach, we performed pull-down experiments. First, we used lysate of p53-null H1299/K3 cells in which endogenous E6AP expression is repressed, but not completely abolished by stable RNA interference [21]. We expressed p53 in these cells and added GST-HIS or GST-HPV16 E6 to the cell lysates, in the absence or presence of either chemically synthesized pep11** or pep11**m. We found that both E6AP and p53 were bound to GST-HPV16 E6 in the absence of the peptides. This indicates that the residual endogenous concentrations of E6AP in H1299/K3 cells were sufficient to support formation of the HPV16 E6 / E6AP / p53 complex. In the presence of pep11**, the amount of HPV16 E6-bound E6AP decreased whereas the level of bound p53 strongly increased (Fig 6A). This was not observed upon adding the HPV16 E6-binding defective pep11**m control. These findings indicate that pep11** can compete for E6AP binding to HPV16 E6, which is linked to strongly increased p53 amounts in the trimeric complex.

To investigate HPV16 E6 / pep11** / p53 complex formation in the absence of E6AP, the H1299/K3 lysate was depleted of E6AP by a pre-clearing step using GST-HPV16 E6 (Fig 6B). GST pull-down experiments from the E6AP-cleared lysate indicate that GST-HPV16 E6 alone does not interact with p53. However, addition of pep11** enabled binding of HPV16 E6 to p53 (Fig 6B), showing that pep11** alone, in the absence of detectable E6AP, is able to efficiently induce the formation of a trimeric complex, together with HPV16 E6 and p53.

## Discussion

The targeted inhibition of E6 could provide an attractive therapeutic strategy for the treatment of HPV-positive tumors. We have previously identified an E6-inhibitory peptide, pep11, and have also developed variants, which specifically bind to the HPV16 E6 protein by targeting the recently identified E6AP binding pocket [16,19]. Compared to the interaction between HPV16 E6 and E6APpep, the binding of pep11 variants is of higher affinity, results in higher increases of p53 levels, and in induction of apoptosis, specifically in HPV16-positive cancer cells [16,19]. The overall aim of this study is to get further insights into the intracellular interaction between HPV16 E6 and E6-binding competent pep11 variants, serving as model for E6 inhibitory molecules.

It has been shown that the binding of E6APpep leads to a conformational change of the HPV16 E6 protein [20,32] which enables binding to p53 [31]. Our results strongly suggest that the interaction of inhibitory pep11 peptides with HPV16 E6 also leads to a conformational change of the E6 protein which induces binding to p53 in a trimeric complex. This is supported by the following findings: (i) HPV16 E6 can bridge E6-binding competent pep11 variants to p53 in cells; (ii) E6-binding competent pep11 variants can bridge HPV16 E6 and p53 in cells; and (iii) E6-binding competent pep11 variants displace E6AP on HPV16 E6 and, by themselves, are sufficient to induce formation of a trimeric complex with HPV16 E6 and p53, in mammalian two hybrid assays and in GST pull-down analyses. Taken together, these results provide the first experimental evidence that E6-targeting molecules, which do not contain the LxxLL sequence of natural interaction partners, are able to efficiently induce complex formation between E6 and p53.

Ectopic co-expression of HPV16 E6 with HPV16 E6-binding competent pep11** led to a translocation of E6 into perinuclear structures which, most likely, are aggresomes. Consistently, immunoblot analyses show that a substantial amount of HPV16 E6 is found in the detergent-insoluble cellular protein fraction. This suggests that the E6-binding competent pep11**, but not the E6-binding defective control pep11**m, induces co-aggregation with E6, upon ectopic co-expression. This notion is supported by results from *in vitro* analyses, indicating that binding of pep11** to HPV16 E6 can lead to ligand-induced E6 aggregation [19]. In addition, we found that pep11** expression led to an increase of overall HPV16 E6 protein levels. This observation is reminiscent of reports describing stabilization of HPV16 E6 by E6APpep [31] or E6AP [30], both of which also target the E6AP binding pocket. Taken together, this set of experiments provides further evidence that pep11** and HPV16 E6 interact intracellularly. Furthermore, the results raise the possibilities that (i) pep11** inactivates E6 to a significant part by forming co-aggregates that are translocated into aggresomes and (ii) pep11** binding to HPV16 E6 leads to substantially elevated HPV16 E6 protein levels.

To address the latter two issues, we expressed E6-binding competent and E6-binding defective pep11** variants in HPV16-positive cells, and monitored changes of endogenous E6 protein. Under these experimental conditions, we did not detect an appreciable translocation of E6 into the detergent insoluble protein fraction. This suggests that the pep11** and pep11’-induced aggresomal translocation is primarily a result of aggregate formation resulting from the high E6 levels that are generated by ectopic expression, but likely does not constitute a mechanism which substantially contributes to E6 inhibition by pep11** and pep11’, at endogenous E6 levels. However, as observed for the ectopic co-expression, the endogenous amounts of HPV16 E6 protein were also clearly augmented in HPV16-positive cells upon intracellular expression of pep11** and pep11’, and not of the corresponding E6-binding defective mutant peptides. Thus, besides mediating a trimeric complex formation together with HPV16 E6 and p53, the E6-binding competent pep11 variants also increase endogenous HPV16 E6 levels.

The presents study raises the possibility that cellular factors, other than proteins containing the LxxLL motif, can mediate the formation of trimeric complexes with E6 and p53. This could provide an explanation for reports describing the E6AP-independent p53 degradation by high risk E6 [9,10] and the E6AP-independent inactivation of p53 by HPV16 E6 in mice [11]. These latter studies also raise the possibility that E6-targeting molecules designed for therapeutic purposes might either lead to the activation or inactivation of p53, by mediating an interaction between high-risk HPV E6 proteins and p53. Together with a potential increase of intracellular E6 levels, this could lead to unpredictable functional consequences.

In the case of the E6-inhibitory pep11 variants, however, the trimeric complex formation, together with E6 and p53, is linked to an increase of overall p53 levels and induction of apoptosis [16], despite of an increase in total E6 protein levels. The following scenario could account for these observations: By targeting the E6AP binding pocket and reshaping of HPV16 E6 [19], the E6-binding competent pep11 variants can stabilize E6, similarly as reported for E6AP or E6APpep [30,31]. Yet, overall p53 levels are augmented since the E6-binding competent pep11 variants induce an efficient sequestration of the limited intracellular E6 quantities into a trimeric complex with p53. Furthermore, the pep11 variants bind to the E6AP pocket of HPV16 E6 and thereby can competitively interfere with E6AP binding. This is supported by the findings that E6-binding competent pep11 variants (i) have a much higher *in vitro* binding affinity for HPV16 E6 than the E6AP interaction domain [16,19], (ii) can restore intracellular p53 protein levels in HPV16-positive cancer cells that endogenously express E6AP [16,19], and (iii) can compete for HPV16 E6 / E6AP binding.

In conclusion, these data indicate that HPV16 E6-targeting molecules, which do not encompass the LxxLL motif, can efficiently induce the interaction of E6 with p53, at the intracellular level. Despite of an overall increase of HPV16 E6 protein levels, this is linked to the reconstitution of p53 and induction of apoptosis in HPV16-positive cancer cells [16,19]. At the mechanistic level, these results show that targeting of HPV16 E6 by molecules designed to contact the E6AP binding pocket can lead to the formation of a trimeric complex with p53, with therapeutically desired functional consequences. These findings are in support of the notion that the E6AP binding pocket could represent an attractive target region for the design of therapeutic E6 inhibitors [19].

## Supporting Information

S1 Fig Comparison of pep11’ and pep11**. (A) Modified amino acid sequence of pep11’ compared to pep11** [16]. Black boxes indicate residues which were exchanged in pep11** to generate pep11’. (B) Intracellular binding analyses. Mammalian two-hybrid assays upon co-expression in HeLa cells of individual peptides linked to GAL4-BD and individual E6 proteins linked to VP16-AD. Both pep11** and pep11’, but not the corresponding mutants pep11**m and pep11’m bind to HPV16 E6. Both pep11** and pep11’ bind to mutant HPV18 E6 proteins R76A and R76S [19], but not wildtype (wt) HPV18 E6. Both pep11** and pep11’ bind to mutant HPV31 E6 W78H, but not mutant HPV31 E6 W78A [19] or wt HPV31 E6. Shown are relative luciferase activities (RLA) of the co-transfected reporter plasmid under transcriptional control of GAL4-binding sites above those of control-transfected cells (expressing the corresponding peptide-GAL4-BD fusions together with VP16AD; values arbitrarily set at 1.0). Results were obtained from three individual experiments, each performed in duplicates. Standard deviations are indicated. (C) Colony formation assays in HPV16-positive SiHa and in HPV-negative U2OS cells, following transfection with different hrGFP-peptide expression vectors, as indicated. hrGFP, negative control (expression vector devoid of peptide sequences).
(TIF)

S2 Fig Immunoblot analyses of endogenous HPV16 E6, upon intracellular expression of E6-targeting peptides in SiHa cells. Expression of hrGFP-linked peptides pep11* [16] or pep11** (both E6-binding competent) and the respective control peptides pep11*m or pep11**m (both E6-binding defective) in HPV-16 positive SiHa cells. Loading of protein extracts was normalized for equal transfection efficiencies, as determined by a co-transfected β-galactosidase expression vector. Expression levels of p53, of the p53-target gene p21, and of individual peptide-hrGFP fusion proteins are indicated. α-Tub, α-tubulin.
(TIF)
