# Supplementary figures and images for: Intracellular Analysis of the Interaction between the Human Papillomavirus Type 16 E6 Oncoprotein and Inhibitory Peptides
Source: PLoS One. 2015 Jul 7;10(7):e0132339. doi: 10.1371/journal.pone.0132339 (PMC4495056; doi:10.1371/journal.pone.0132339)

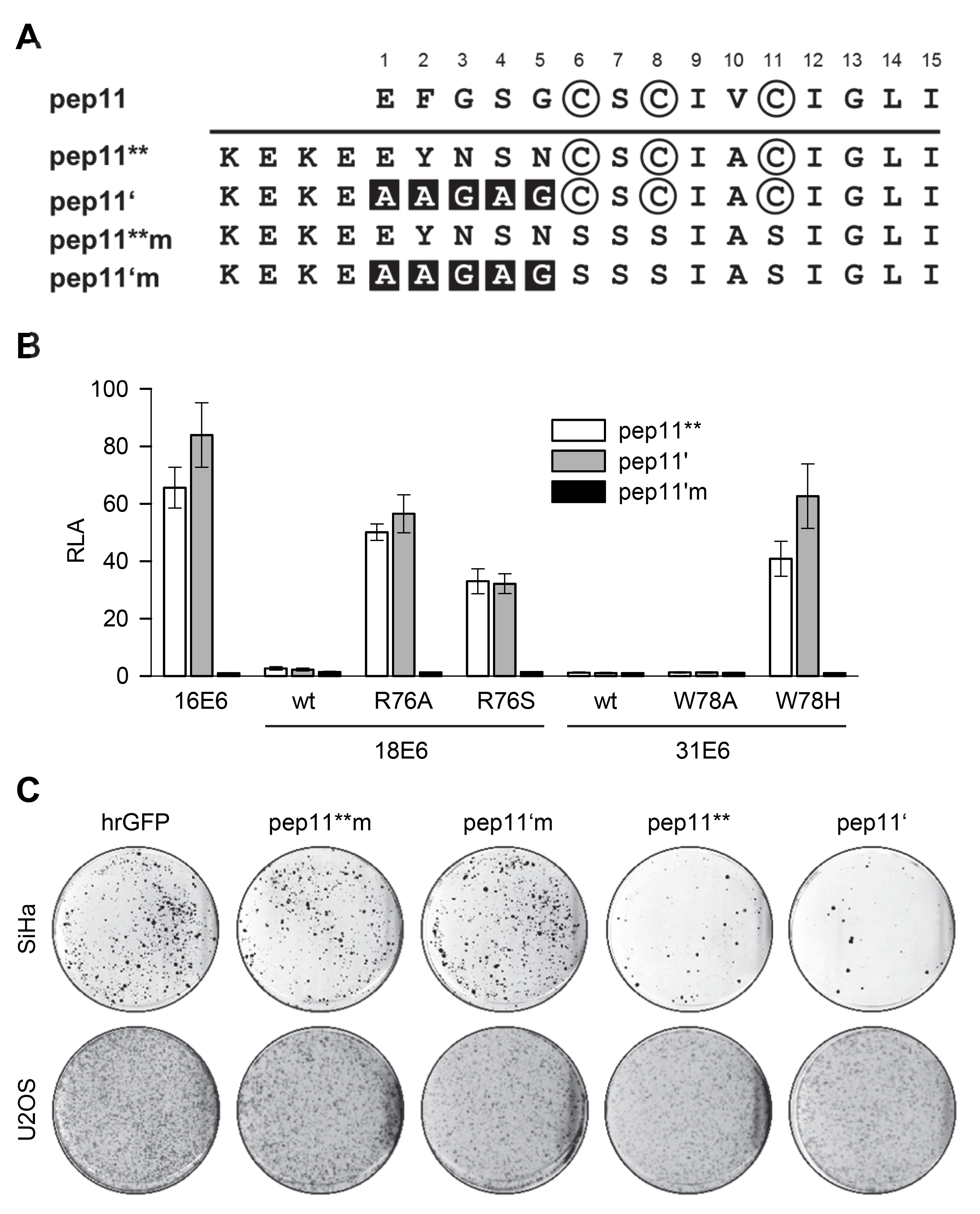

Supplement: S1 Fig — (A) Modified amino acid sequence of pep11’ compared to pep11** [16]. Black boxes indicate residues which were exchanged in pep11** to generate pep11’. (B) Intracellular binding analyses. Mammalian two-hybrid assays upon co-expression in HeLa cells of individual peptides linked to GAL4-BD and individual E6 proteins linked to VP16-AD. Both pep11** and pep11’, but not the corresponding mutants pep11**m and pep11’m bind to HPV16 E6. Both pep11** and pep11’ bind to mutant HPV18 E6 proteins R76A and R76S [19], but not wildtype (wt) HPV18 E6. Both pep11** and pep11’ bind to mutant HPV31 E6 W78H, but not mutant HPV31 E6 W78A [19] or wt HPV31 E6. Shown are relative luciferase activities (RLA) of the co-transfected reporter plasmid under transcriptional control of GAL4-binding sites above those of control-transfected cells (expressing the corresponding peptide-GAL4-BD fusions together with VP16AD; values arbitrarily set at 1.0). Results were obtained from three individual experiments, each performed in duplicates. Standard deviations are indicated. (C) Colony formation assays in HPV16-positive SiHa and in HPV-negative U2OS cells, following transfection with different hrGFP-peptide expression vectors, as indicated. hrGFP, negative control (expression vector devoid of peptide sequences). (TIF) [file pone.0132339.s001.tif]

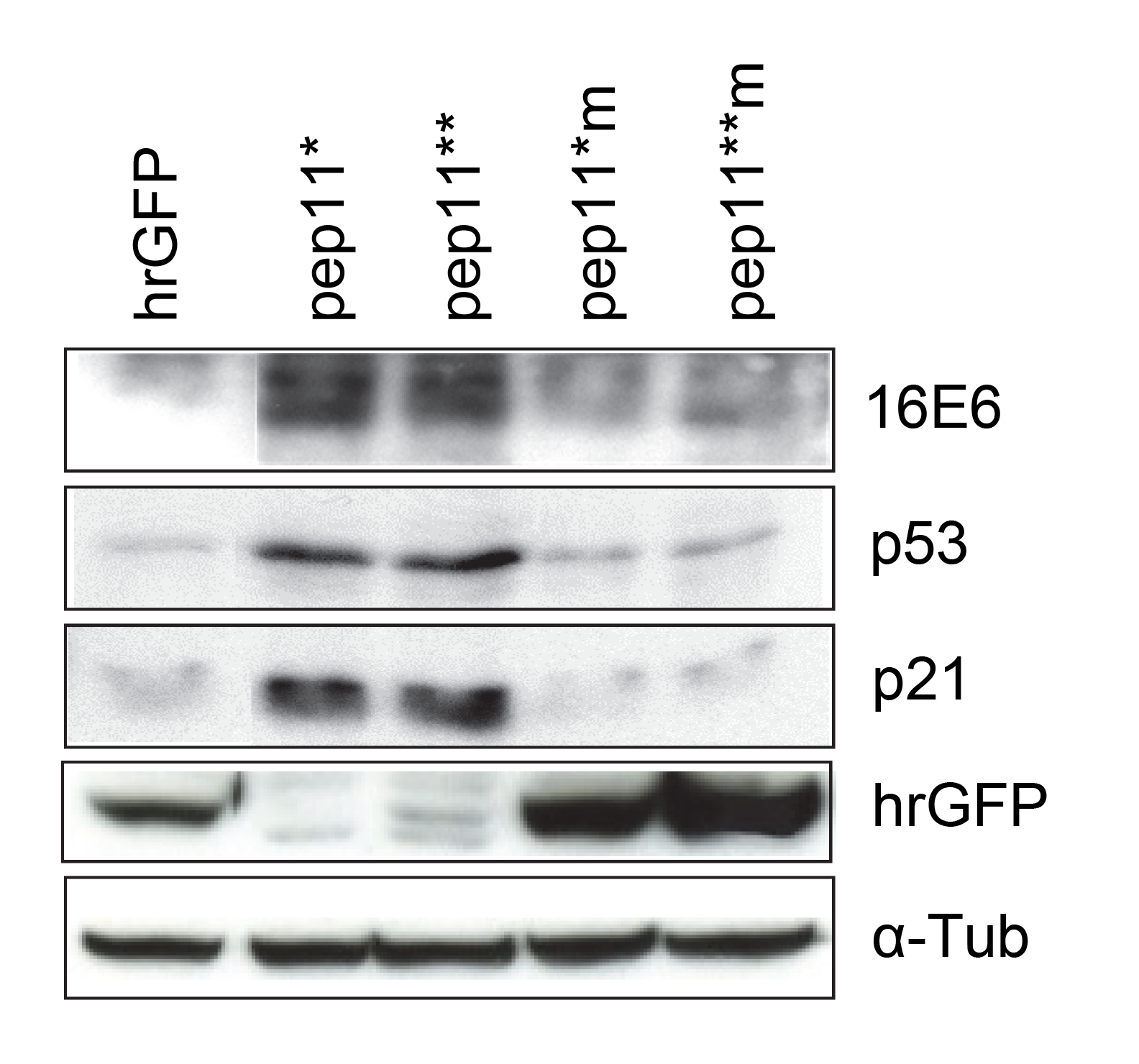

Supplement: S2 Fig — Expression of hrGFP-linked peptides pep11* [16] or pep11** (both E6-binding competent) and the respective control peptides pep11*m or pep11**m (both E6-binding defective) in HPV-16 positive SiHa cells. Loading of protein extracts was normalized for equal transfection efficiencies, as determined by a co-transfected β-galactosidase expression vector. Expression levels of p53, of the p53-target gene p21, and of individual peptide-hrGFP fusion proteins are indicated. α-Tub, α-tubulin. (TIF) [file pone.0132339.s002.tif]
